# The Effect of Radiofrequency Exposure on the Cytotoxic Activity of Sunitinib and a Novel Sunitinib-Class Compound 4, in Colorectal Cancer Cells

**DOI:** 10.3390/ijms27135953

**Published:** 2026-07-02

**Authors:** Ayse Erol Bozkurt, Mediha Suleymanoglu, Tugce Cinek, Nilgun Karali, Sedat Ballikaya, Mehmet Salih Iyikesici, Serap Kuruca

**Affiliations:** 1 Department of Medical Biology, Istanbul Faculty of Medicine, Istanbul University, 34093 Istanbul, Turkey; ayse.erol@istanbul.edu.tr (A.E.B.); mediha.suleymanoglu@istanbul.edu.tr (M.S.); 2 Department of Pharmaceutical Chemistry, Faculty of Pharmacy, Istanbul Health and Technology University, 34275 Istanbul, Turkey; tugce.cinek@istun.edu.tr; 3 Department of Pharmaceutical Chemistry, Faculty of Pharmacy, Istanbul University, 34116 Istanbul, Turkey; karalin@istanbul.edu.tr; 4 Department of Engineering Sciences, Faculty of Engineering, Istanbul University–Cerrahpaşa, 34320 Istanbul, Turkey; ballikaya@iuc.edu.tr; 5 Department of Medical Oncology, School of Medicine, Altinbas University, 34147 Istanbul, Turkey; mehmet.iyikesici@altinbas.edu.tr; 6 ChemoThermia Oncology Center, 34295 Istanbul, Turkey; 7 Department of Physiology, Faculty of Medicine, Istanbul Atlas University, 34408 Istanbul, Turkey

**Keywords:** colorectal cancer, radiofrequency hyperthermia, cell viability, tyrosine kinase inhibitor, 2-indolinone, sunitinib

## Abstract

Achieving optimal therapeutic efficacy in colorectal cancer (CRC) remains challenging, highlighting the need for novel treatment strategies. This study evaluated whether radiofrequency (RF) hyperthermia enhances the anticancer effects of sunitinib malate and a newly synthesized tyrosine kinase (TK) inhibitor (compound **4**). HCT116 CRC cells and BEAS-2B normal epithelial cells were treated with increasing concentrations of both agents in the presence or absence of RF exposure using a mobile RF device. Cell viability and death were assessed by MTT assay and flow cytometry (Annexin V/PI). RF exposure was associated with reduced viability and increased cell death in HCT116 cells (*p* < 0.05). Flow cytometric analysis confirmed that RF exposure may contribute to the observed reduction in viability. Notably, the findings suggest a possible beneficial contribution of RF hyperthermia to the observed cytotoxic effects, particularly near the IC50 concentration. In contrast, BEAS-2B cells maintained high viability with no significant changes (*p* > 0.05). These findings suggest that RF exposure may contribute to the observed cytotoxic responses in CRC cells while exerting limited effects on non-malignant cells, suggesting a promising strategy for targeted therapy.

## 1. Introduction

Colorectal cancer (CRC) is a major global health issue, ranking as the third most frequently diagnosed malignancy and accounting for nearly 10% of all cancer cases worldwide [1,2]. Current treatment options for CRC primarily include surgical resection, chemotherapy, radiotherapy, and targeted therapies. Despite these established modalities, achieving optimal anticancer efficacy remains challenging, highlighting the urgent need for innovative therapeutic strategies. In particular, the development of novel agents and rational combination regimens targeting critical molecular pathways is essential. Conventional approaches, such as chemotherapy and radiotherapy, are limited by their lack of specificity, affecting not only malignant cells but also healthy tissues. This nonspecific toxicity often results in debilitating side effects, including alopecia, fatigue, and other complications [3,4,5,6]. Consequently, there is a pressing demand for next-generation therapies that can selectively eradicate cancer cells while minimizing adverse effects on normal tissues.

**Figure 1:**
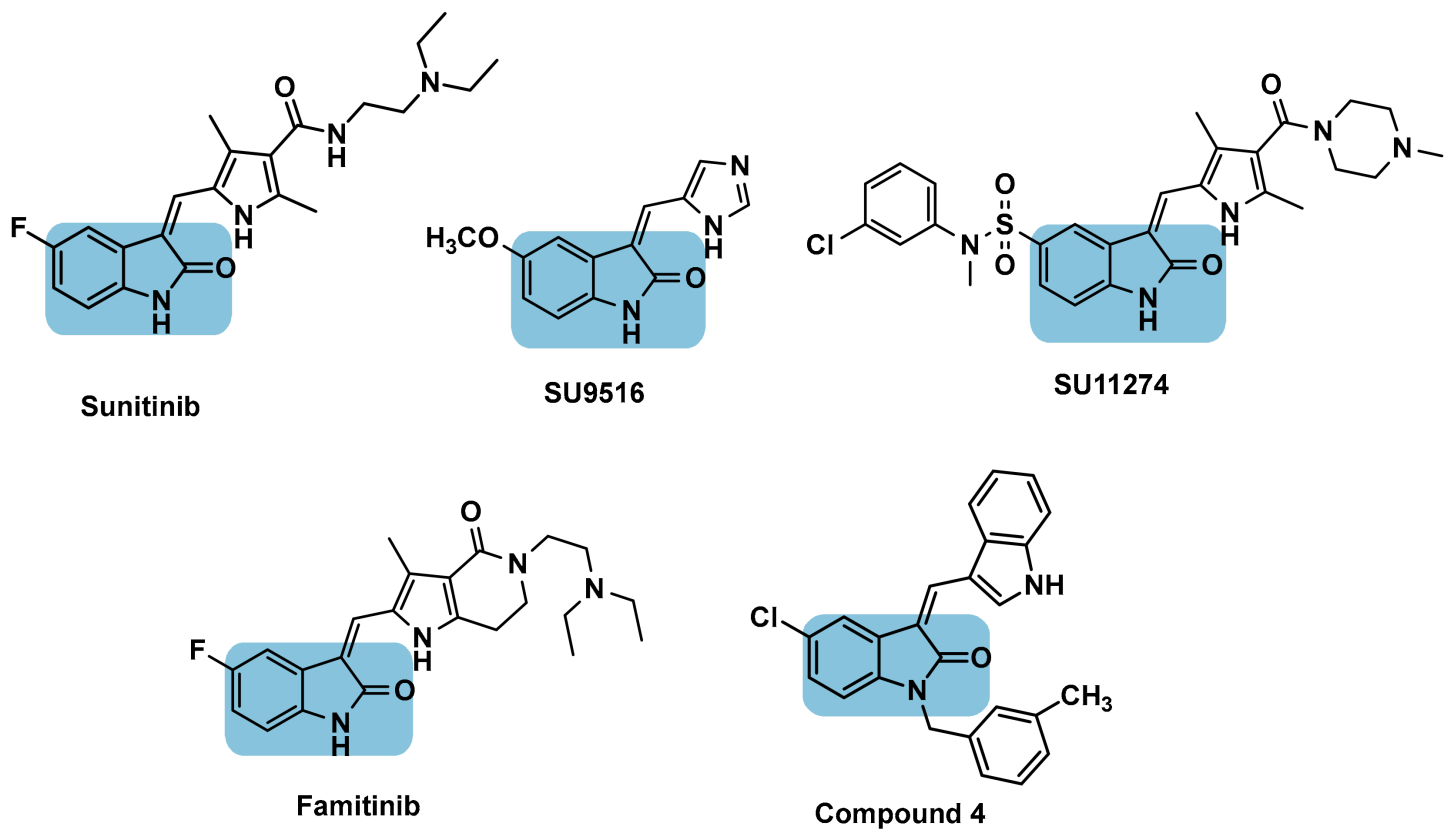
Structural formulas of some anticancer agents and compound **4** bearing 2-indolinone skeleton.

Immunotherapy and targeted therapies, such as tyrosine kinase (TK) inhibitors, are currently being evaluated in clinical trials for patients with CRC [7]. Among these therapies, one of the clinically approved and extensively investigated agents is sunitinib, a TK inhibitor. Sunitinib malate is the first 2-indolinone derivative multitargeted TK inhibitor approved for clinical use, and it is an orally administered multikinase inhibitor that targets several receptors, including vascular endothelial growth factor receptors (VEGFRs), platelet-derived growth factor receptors (PDGFR-α and PDGFR-β), c-KIT, and additional TKs (Figure 1). Ongoing clinical trials continue to evaluate the therapeutic potential of sunitinib in a range of other malignancies, including metastatic breast, colorectal, and lung cancers [8,9,10]. However, its efficacy remains relatively limited. Therefore, researchers have sought new approaches that could complement chemotherapy, radiotherapy, and targeted therapies while minimizing damage to healthy cells.

Newly synthesized compounds with chemical structures similar to sunitinib, which possess TK inhibitory activity, are providing novel approaches for CRC treatment. SU9516 is a selective cyclin-dependent kinase 2 inhibitor bearing an imidazole ring at the 3-position of the 2-indolinone scaffold [11]. A study conducted in 2017 reported that SU9516 increased the sensitivity of ABT-737–resistant Caco-2 colorectal carcinoma cells to ABT-737 [12]. SU11274 is another 2-indolinone TK inhibitor that targets c-Met kinase [13]. SU11274 was found to significantly reduce the number of LoVo cells in the S and G2 phases. Moreover, treatment of LoVo colorectal carcinoma cells with SU11274 suppressed cell growth and proliferation through the inhibition of both c-Met phosphorylation and proliferative signaling [14]. Famitinib, which is a sunitinib analog, has been shown to exert broad TK inhibitory activity by potently inhibiting VEGFR-2 and -3, PDGFR, c-KIT, and RET, thereby demonstrating cytotoxic effects against various solid tumors. Clinical investigations, including phase II studies, have evaluated famitinib in several malignancies such as CRC and triple-negative breast cancer [15,16,17,18,19] (Figure 1).

The pyrrole ring present in the sunitinib structure was replaced with an indole ring, leading to the design of 2-indolinone-indole hybrid compounds. Indole is an important structural motif frequently employed in the design of anticancer agents. The ability of indole derivatives to interact with various cancer-related targets, such as protein kinases, tubulin, and apoptosis-regulating proteins, has led to the discovery of numerous compounds with promising anticancer activity [20,21]. Accordingly, 2-indolinone-indole hybrid compounds have also been reported as promising anticancer agents against various cancer cell lines [22,23]. In studies in which a series of indole-2-indolinone hybrids bearing benzyl or benzoyl substituents at the 1-position of the 2-indolinone ring were designed and synthesized, it was determined that these hybrid compounds exhibited stronger cytotoxic potency, lower toxicity, and superior selectivity indices than sunitinib malate. In addition, these compounds induced apoptosis and inhibited the ERK signaling pathway, had strong c-SRC, PDGF-β and c-Met kinase inhibitory effects [9,24].

Radiofrequency (RF)-based hyperthermia has been shown to sensitize tumor cells to chemotherapy by enhancing drug uptake, altering cell membrane permeability, generating reactive oxygen species, and inducing apoptosis [25,26,27]. RF-based hyperthermia is particularly advantageous due to its superior tissue penetration and its relatively low effect on healthy tissue at a fixed frequency of 13.56 MHz. Although RF has been combined with conventional drugs such as sunitinib or newly synthesized compounds with synergistic results [28,29], limited data exist regarding its combination with TK inhibitors.

The compounds developed following our group’s previous design strategy represent a novel modification distinct from sunitinib and its derivative TK inhibitor compounds. The most important differences in compound **4** in the present article are that the 2-indolinone scaffold carries a benzyl substituent at the 1-position, an indole ring instead of a pyrrole residue at the 3-position, and a chlorine group at the 5-position. A series of 1-benzyl-2-indolinone indole hybrid compounds bearing these structural differences were designed and synthesized in study of our research group, and their cytotoxicities and mechanisms of action were examined. The results obtained showed that these compounds had strong selective effects on colon HCT-116 and pancreatic MIA PaCa-2 cancer cells, induced apoptosis, reduced ERK signaling, and inhibited PDGFR-β, SRC, c-MET signalings. The hybrid compounds were found to exhibit significantly higher inhibitory effects and safety profiles when compared to sunitinib malate [24].

This study aimed to investigate the cytotoxic effects of sunitinib malate and a newly synthesized TK inhibitor, the novel 1-(3-methylbenzyl)-5-chloro-2-indolinone indole hybrid (compound **4**), on HCT116 colorectal carcinoma cells and BEAS-2B normal epithelial cells in both the presence and absence of RF exposure. By comparing the ability of RF hyperthermia to potentiate the cytotoxic effects of sunitinib malate and compound **4**, this research also aimed to provide valuable insights for the development and discovery of novel hybrid anticancer compounds (Figure 1).

## 2. Results

### 2.1. Chemistry

**Scheme 1:**
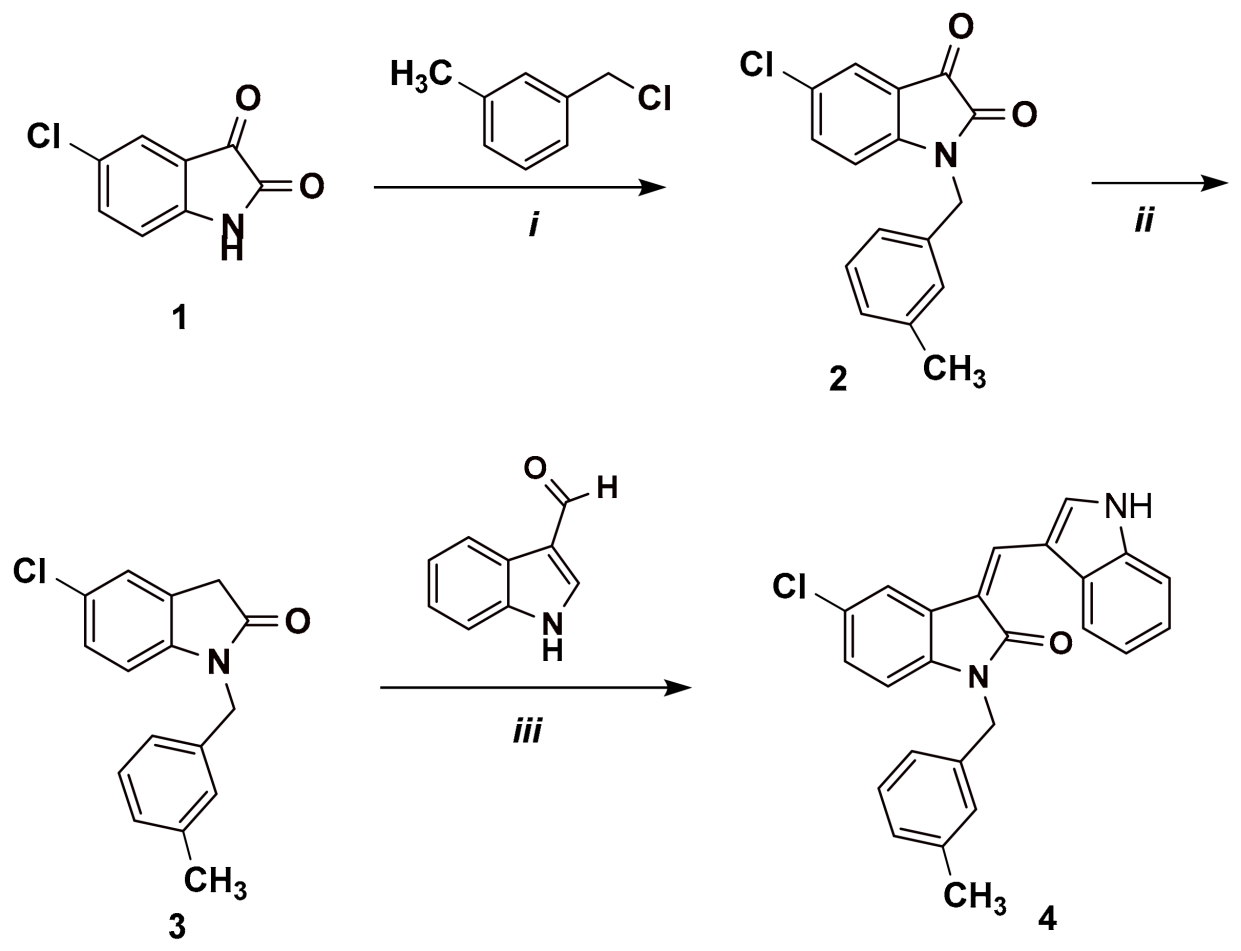
Synthesis of 1-(3-methylbenzyl)-3-[(1*H*-indole-3-yl)methylidene]-5-chloro-1,3-dihydro-2*H*-indole-2-one (**4**). Reagents and conditions: (i) K_2_CO_3_, KI, DMF, 50–60 °C, reflux, 2 h; (ii) H_2_NNH_2_, 120–130 °C, reflux, 4 h; (iii) piperidine, EtOH, 80–90 °C, reflux, 5 h.

For the synthesis of compound **4**, 5-chloro-1*H*-indole-2,3-dione (**1**) was reacted with 3-methylbenzyl chloride in the presence of potassium carbonate and potassium iodide to obtain 1-(3-methlybenzyl 5-chloro-1*H*-indole-2,3-dione (**2**). Subsequently, compound 2 was reduced according to the Wolff-Kischer reduction by the use of hydrazine hydrate and this reaction converted the carbonyl group into methylene moiety under high-temperature conditions and afforded the corresponding 1-(3-methlybenzyl)-5-chloro-1,3-dihydro-2*H*-indol-2-one (**3**) derivative. Finally, the desired compound 1-(3-methylbenzyl)-3-(1*H*-indole-3-ylmethylidene)-5-chloro-1,3-dihydro-2*H*-indol-2-one (**4**) was synthesized by the condensation of compound **3** and 1*H*-indole-3-carbaldehyde (Scheme 1). The structure and purity of compound **4** were characterized by IR, ^1^H NMR, ^13^C NMR-APT, and LC-MS analysis.

In the IR spectrum of compound **4**, the characteristic N–H and C=O stretching bands were observed at 3269 and 1662 cm^−1^, respectively (Figure S1). In the ^1^H NMR spectrum of compound **4**, singlet signals corresponding to the indole NH and vinylic protons, appearing at δ 12.24 and δ 8.42 ppm, respectively (Figures S2 and S3). These signals were confirmed by the 2-indolinone indole hybrid structure. The ^13^C NMR spectral data were interpreted based on APT experiments. The carbonyl (C=O) carbon of the 2-indolinone ring appeared in the downfield region at δ 166.49 ppm. Additionally, the presence of upfield resonance corresponding to benzylic (–CH_2_–) carbon at δ 43.05 ppm provided further confirmation of the structure of the synthesized compound **4** (Figures S4 and S5). Mass spectral analyses of compound **4** were performed under negative-ion electrospray ionization (ESI-) conditions. The detected deprotonated molecular ion [M-H]^−^ in the ESI-MS spectrum was consistent with the calculated molecular weights of the corresponding compound (Figure S6). Furthermore, the appearance of [(M-H)+2]^−^ isotope peak in the spectrum due to the presence of the ^37^Cl isotope provided additional evidence supporting the proposed structure.

### 2.2. In Vitro Biological Studies

#### 2.2.1. Measurement of the Hyperthermia Effect of the Radiofrequency Generator and Evaluation Results

Cell lines obtained from ATCC were expanded, cryopreserved, and stored in liquid nitrogen tanks for the standardization studies measuring RF efficacy under in vitro conditions. HCT116 human colon cancer cell line and the BEAS-2B human bronchial epithelial cell line were rapidly thawed from liquid nitrogen, washed with medium, and transferred to culture conditions. The cells were cultured in 75 cm^2^ flasks and passaged every three days prior to experimentation.

**Figure 2:**
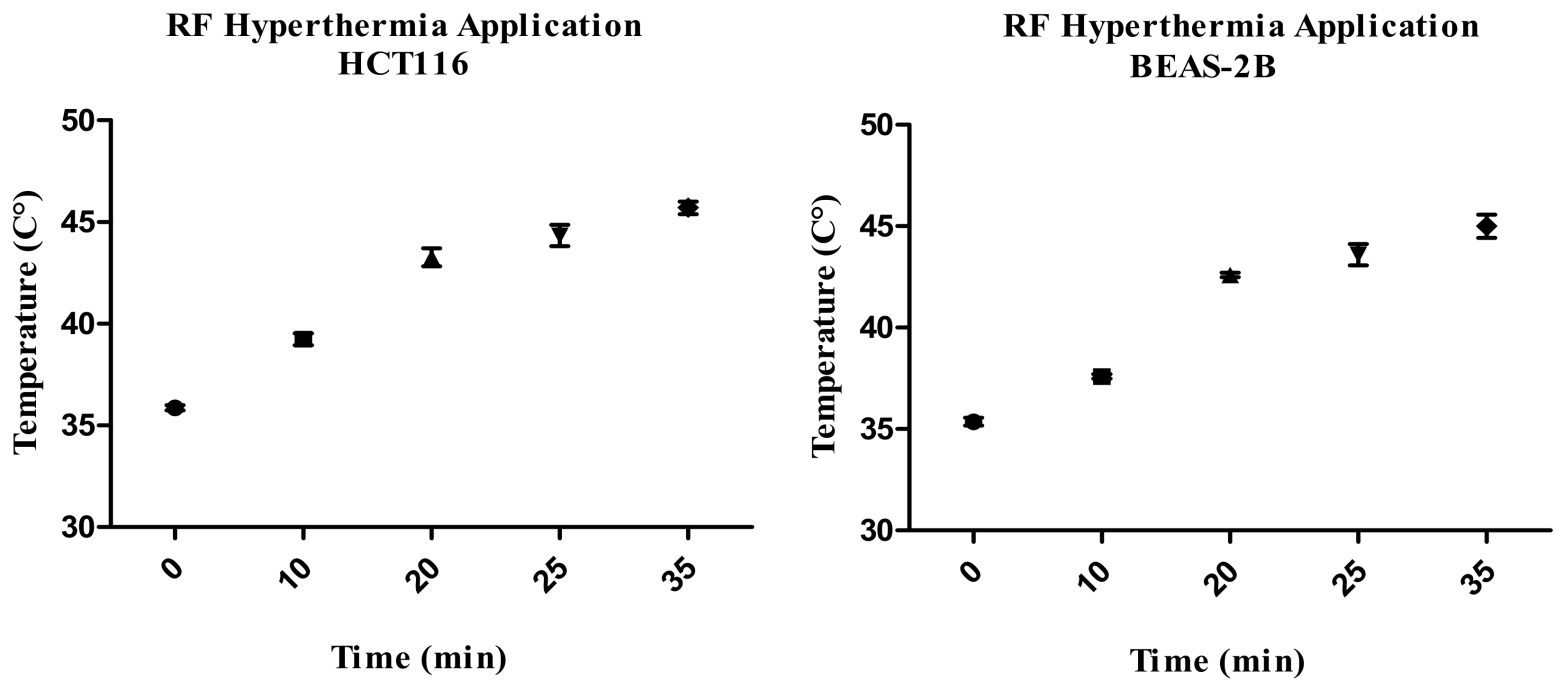
Time-dependent changes in temperature measurements following RF application (75 W-13.56 MHz) in HCT116 and BEAS-2B cells.

Time-dependent temperature measurements following RF exposure (75 W, 13.56 MHz) demonstrated a gradual increase in temperature in both HCT116 and BEAS-2B cells, reaching the hyperthermic range. The thermal profiles were highly similar between the two cell lines, with no notable differences in temperature elevation throughout the exposure period (Figure 2).

As shown in Figure 2, a transient temperature overshoot of up to 45 °C was observed during the initial heating phase, after which the system stabilized and maintained the target temperature of 42–43 °C for the remainder of the exposure time.

#### 2.2.2. Cell Cytotoxicity Effects After Combination Treatment

The cytotoxic and anti-proliferative effects of the newly synthesized compound **4** and the reference drug sunitinib were assessed in colon cancer and non-cancerous cell lines using the MTT assay, with and without RF-induced hyperthermia. RF treatment was applied in five different concentrations (0.625, 1.25, 2.5, 5.0, or 10.0 µM) of each compound to determine the impact of hyperthermia on cell viability. The absorbance values obtained from the MTT assay revealed concentration-dependent responses in both cell lines. Comparative evaluation indicated that sunitinib malate and compound **4** exhibited distinct inhibitory activity, and sunitinib was therefore used as the reference standard due to its structural similarity to compound **4** and its well-established cytotoxic potential. RF exposure was associated with lower absorbance values in HCT116 cells.

**Figure 3:**
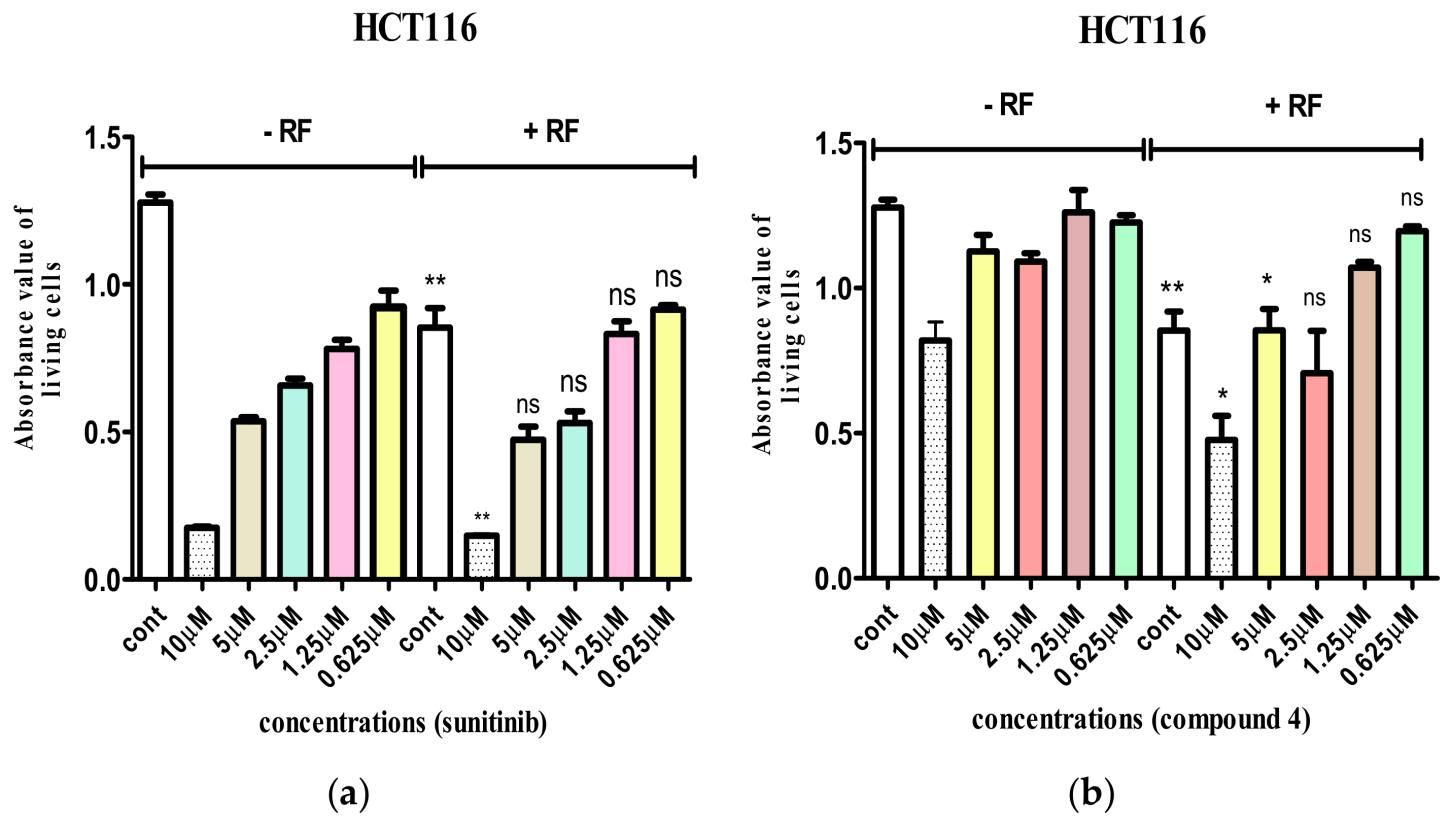
Effect of sunitinib treatment with and without RF hyperthermia (**a**); effect of compound **4** and RF hyperthermia on HCT116 cell viability determined by MTT assay (**b**) in HCT116 cells. The non-RF-treated group is presented on the left, whereas the RF-treated group is shown on the right in each graph (−RF: non RF treated; + RF: RF treated). Data are presented as mean ± SEM (n = 3 biological replicates). Statistical significance was determined using Student’s *t*-test for comparisons between two groups. * *p* < 0.05 and ** *p* < 0.01 indicate significant differences between the RF-treated and non-RF-treated groups at the same concentration; ns, not significant.

**Figure 4:**
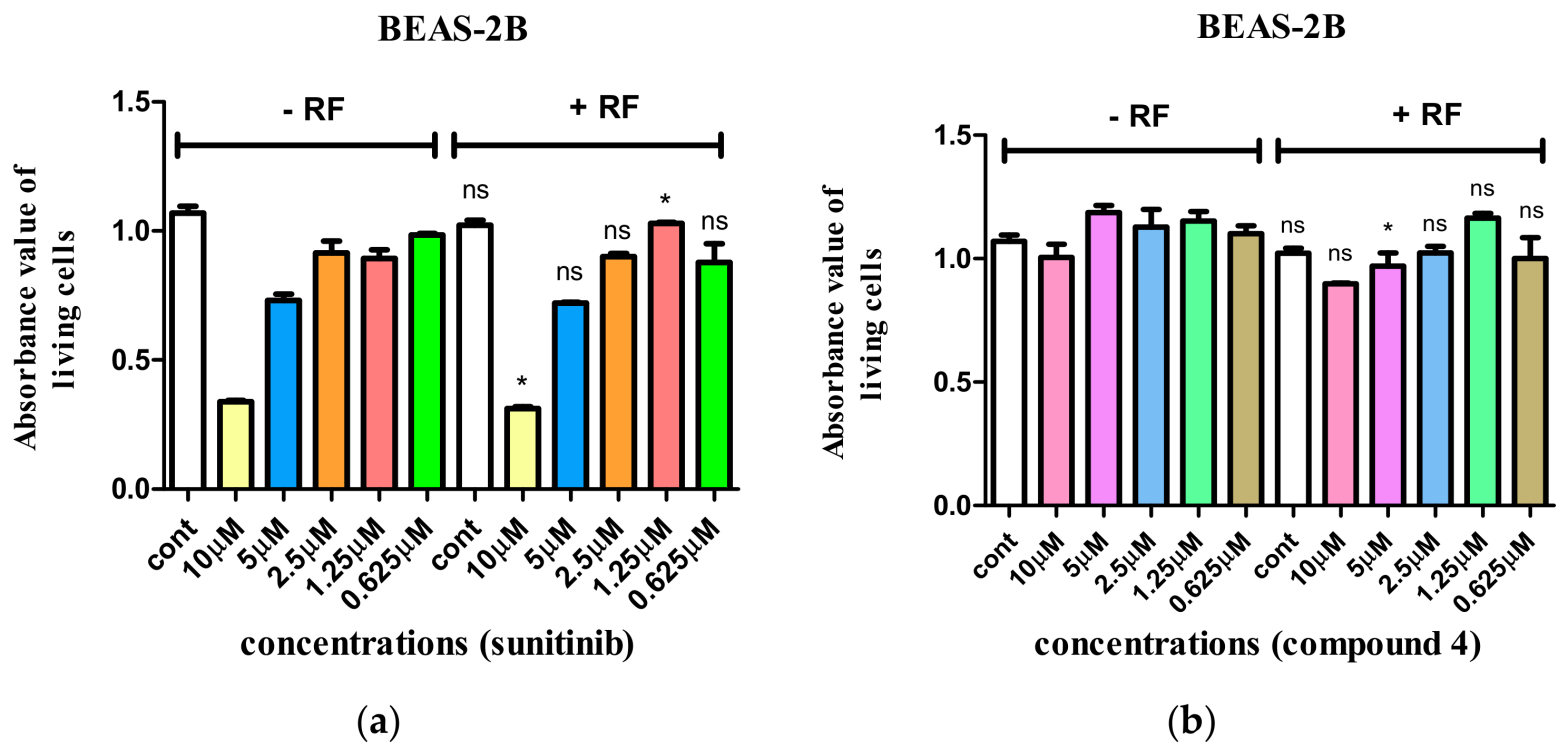
Effects of sunitinib treatment with and without RF hyperthermia (**a**); effect of compound **4** and RF hyperthermia on BEAS-2B cell viability determined by MTT assay (**b**) in BEAS-2B cells. The non-RF-treated group is presented on the left, whereas the RF-treated group is shown on the right in each graphs (−RF: non RF treated; + RF: RF treated). Data are presented as mean ± SEM (n = 3 biological replicates). Statistical significance was determined using Student’s *t*-test for comparisons between two groups. * *p* < 0.05 indicate significant differences between the RF-treated and non-RF-treated groups at the same concentration; ns, not significant.

A dose-dependent decrease in absorbance values was observed in HCT116 cells following sunitinib malate treatment (Figure 3a), with higher concentrations resulting in lower absorbance values. In BEAS-2B cells, although a slight reduction in absorbance was observed at higher concentrations (Figure 4a), cell viability remained relatively preserved. Similarly, compound **4** exhibited a dose-dependent cytotoxic effect in HCT116 cells (Figure 3b), whereas no apparent cytotoxic effect was observed in BEAS-2B cells (Figure 4b). Furthermore, at higher concentrations (5 and 10 μM), compound **4** tended to exhibit lower absorbance values than sunitinib in HCT116 cells. In HCT116 cells, RF exposure was associated with lower absorbance values in compound **4**-treated cells compared with the corresponding non-RF-treated groups at the same concentrations. A similar pattern was observed in the sunitinib-treated group, where absorbance values were also reduced in the presence of RF hyperthermia (Figure 3). In contrast, BEAS-2B cells exhibited only limited and concentration-dependent changes following RF exposure in combination with compound **4** or sunitinib malate. Although statistically significant differences were observed at selected concentrations, the overall response was less pronounced than that observed in HCT116 cells. These findings suggest a differential response between the cancerous and non-malignant cell lines under the experimental conditions employed (Figure 4).

#### 2.2.3. Flow Cytometric Evaluation of Cell Viability and Cell Death Following RF and Compound **4** Treatment

**Figure 5:**
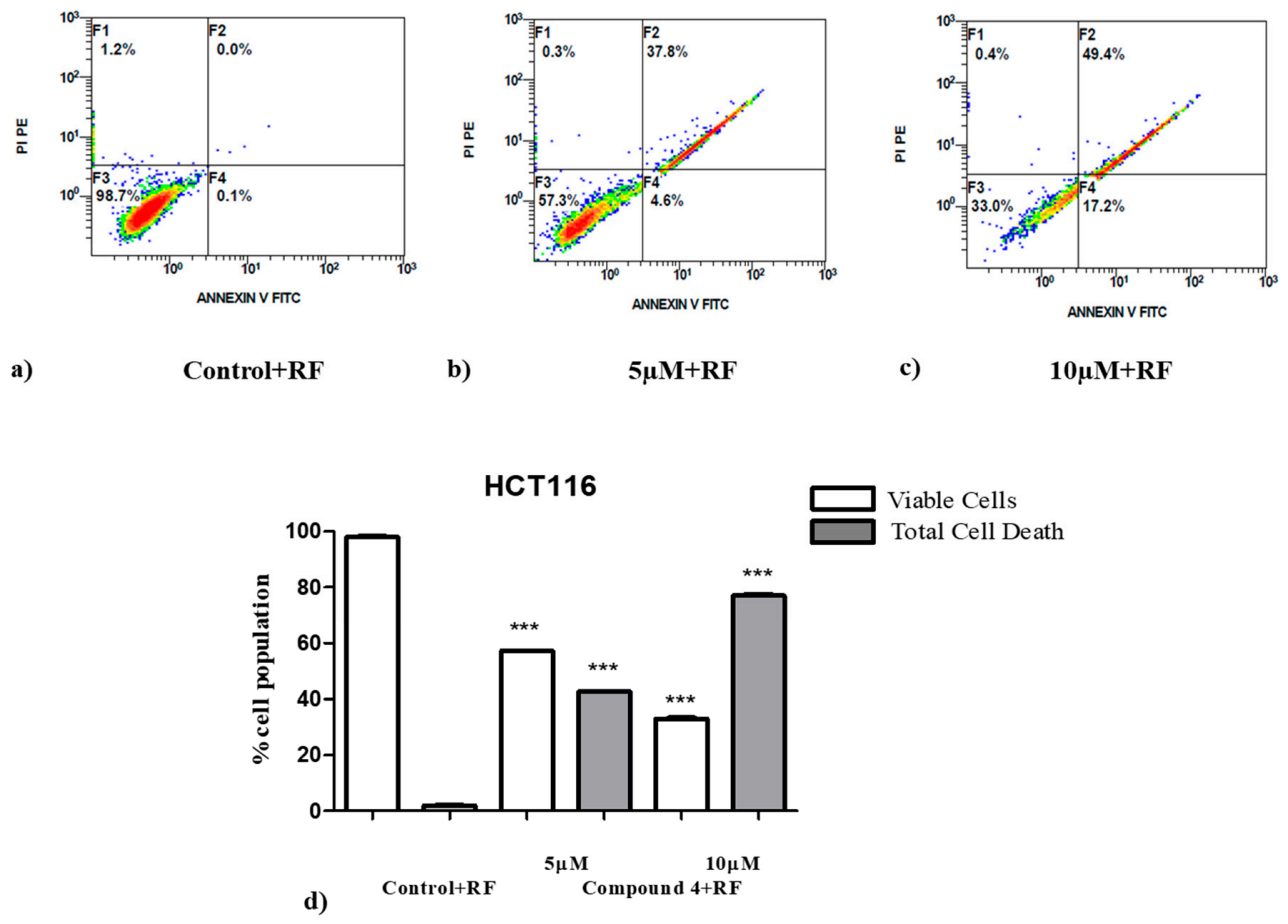
Comparative flow cytometric analysis of viability and cell death levels induced by compound **4** with RF Hyperthermia exposure in HCT116 cells. (**a**) Flow cytometry analysis graph: Control group with RF hyperthermia (**b**) Flow cytometry analysis graph: Compound **4** (5 µM concentration) with RF hyperthermia (**c**) Flow cytometry analysis graph: Compound **4** (10 µM IC_50_ concentration) with RF hyperthermia (**d**) Comparative statistical analysis between all groups. -Data are presented as mean ± SEM (n = 3 biological replicates). Statistical significance was determined using Student’s *t*-test for comparisons between three groups. *** *p* < 0.0001 vs. control group.

Flow cytometric analysis revealed that RF exposure alone had minimal impact on cell viability in HCT116 cells. In contrast, co-treatment with compound **4** and RF resulted in a marked reduction in viability and a corresponding increase in total cell death in a dose-dependent manner. Notably, treatment with 5 µM (approximately half of the IC_50_ concentration) in combination with RF, reduced cell viability to around 50%. Furthermore, at the approximate IC_50_ concentration (10 µM), the addition of RF led to a pronounced decrease in viability well below 50%, indicating increased cell death under combined treatment conditions. Flow cytometric findings showed increased cell death and reduced viability in cells treated with compound **4** under RF exposure (Figure 5).

## 3. Discussion

CRC is a common global malignancy, representing the third most diagnosed cancer and about 10% of all cases. Standard treatments, including surgery, chemotherapy, radiotherapy, and targeted therapies, often face limitations in achieving optimal efficacy, highlighting the need for novel therapeutic agents and combination strategies targeting key molecular pathways [1]. In cancer treatment, the primary therapeutic modalities include surgery, radiotherapy, chemotherapy, immunotherapy, targeted therapy, hormone therapy, stem cell transplantation, and precision medicine. These treatments may lead to a range of adverse effects, such as anemia, loss of appetite, bleeding, bruising (thrombocytopenia), constipation, delirium, diarrhea, edema, and fatigue [30].

The application of hyperthermia must remain within safe temperature ranges (39–45 °C), ensuring that cancer cells are effectively eradicated while avoiding damage to healthy cells. At these temperature levels, cells exhibit increased ROS generation, mitochondrial membrane depolarization, and activation of caspase-3 and caspase-9, ultimately leading to DNA fragmentation. Sublethal heating also triggers the expression of heat shock proteins (HSP70 and HSP90), which enhances cellular sensitivity to both chemotherapy and radiotherapy. Activation of the UPR leads to the overexpression of heat shock proteins (HSPs), including HSP70 on the cell membrane, as well as tumor-specific antigens (TSAs) within cancer cells [31,32,33]. The increased presence of HSP70 on the tumor cell surface enhances immune recognition, particularly by natural killer (NK) cells. In addition, tumor cells undergoing necrosis release TSAs and TSA–HSP complexes, which further promote immune cell activation. Drug-mediated hyperthermia can be achieved through several modalities, with recent studies highlighting nanoparticle-based approaches as the predominant strategy. In general, these methods involve delivering and accumulating nanoparticles within the tumor site, followed by targeted irradiation. The interaction of the nanoparticles with the applied external energy subsequently induces localized hyperthermia in the designated region. Although these approaches have the potential to increase intracellular temperature to levels that induce cell death, they may also trigger adverse side effects such as ionization of genetic material or non-selective activity during microwave or radiation-based treatments, which can negatively impact surrounding healthy tissues. This challenge has prompted the investigation of novel strategies capable of elevating the temperature of damaged tissues while simultaneously preserving the integrity of adjacent healthy tissues [34,35].

In recent studies, particular emphasis has been placed on magnetic nanoparticles and treatments that become more effective upon cellular uptake facilitated by heat (hyperthermia) activation [36,37]. RF represents an alternating electromagnetic field that induces oscillation of ions both within the culture medium and intracellular environment, thereby generating heat through ionic motion. The magnitude of the RF-induced thermal effect is governed by several parameters, including the intracellular ion concentration, the ionic composition of the surrounding medium, and the thermal conductivity of the system [29].

Hyperthermia has been widely investigated as an adjuvant strategy to enhance the efficacy of anticancer therapies. Previous studies have demonstrated that hyperthermia can potentiate the cytotoxic effects of chemotherapeutic and targeted agents through multiple mechanisms, including increased cellular drug uptake, alteration of tumor microenvironment, and sensitization of cancer cells to therapeutic stress. For instance, a pilot clinical study reported that the combination of RF hyperthermia with the TK inhibitor gefitinib improved treatment outcomes in patients with advanced non-small cell lung cancer [38]. Similarly, an in vitro study demonstrated that sunitinib malate exhibited enhanced cytotoxic activity when combined with hyperthermia in MCF-7 breast cancer cells [39]. Consistent with these findings, our results suggest that RF exposure may enhance the cytotoxic effects of both sunitinib malate and the newly synthesized TK inhibitor compound **4** in CRC cells. Importantly, our study further extends these observations by evaluating the potential of RF-induced hyperthermia in combination with a newly synthesized TK inhibitor, providing preliminary insights into the possible therapeutic benefit of this combined approach.

However, in this study, we introduced a novel approach by investigating whether hyperthermia could enhance the therapeutic efficacy of sunitinib—a TK inhibitor widely used in cancer treatment—in combination with a newly synthesized compound (compound **4**). Although compound **4** shares the 3-arylidene-2-indolinone scaffold with sunitinib, several structural differences clearly distinguish the two molecules. In our compound, the 2-indolinone ring is 3-methylbenzyl-substituted at the 1-position, the 2-indolinone core carries a chloro group at the 5-position, and an indole ring is present at the 3-position instead of the pyrrole moiety found in sunitinib. The induction of cancer cell death through RF exposure combined with pharmacological agents requires the fulfillment of two key conditions: (i) the agents must demonstrate sufficient cytotoxic activity at clinically relevant concentrations, and (ii) the therapeutic temperature must be carefully controlled to prevent nonspecific necrotic cell death caused by excessive heating. The present study demonstrated the cytotoxic effects of the combined application of compound **4**, RF hyperthermia, and sunitinib malate. Our findings suggest that RF exposure may contribute to the observed cytotoxic response of compound **4**, supporting its potential as an adjunct therapeutic approach. RF exposure was associated with lower absorbance values in HCT116 cells treated with compound **4** and sunitinib. In contrast, BEAS-2B epithelial cells exhibited a more limited response under the same experimental conditions. The inclusion of BEAS-2B cells was intended to provide a comparison between malignant and non-malignant cell lines and to assess the general cellular response to the combined treatment approach. However, because BEAS-2B cells are derived from bronchial epithelium rather than normal colonic tissue, these findings should not be interpreted as evidence of CRC-specific selectivity. Instead, the results indicate that the RF-assisted treatment approach was more effective in HCT116 cells than in the non-malignant epithelial cell line evaluated in this study under in vitro conditions. The magnitude of the RF-induced thermal effect is governed by several parameters, including intracellular ion concentration, the ionic composition of the surrounding medium, and the thermal conductivity of the system [40]. In line with these findings, flow cytometric analysis confirmed that RF exposure may be associated with the cytotoxic effect of compound **4** at the cellular level. The combination treatment led to a dose-dependent decrease in cell viability accompanied by an increase in total cell death. Notably, co-treatment with RF and a sub-IC_50_ concentration of compound **4** reduced cell viability to approximately 50%, while treatment at the IC_50_ concentration resulted in a markedly greater loss of viability. These findings are consistent with the MTT assay results, which also demonstrated a dose-dependent reduction in cell viability that was further enhanced by RF exposure, thereby confirming the cytotoxic potentiation effect across independent experimental approaches.

While our current in vitro results provide a valuable proof-of-concept for RF-assisted chemotherapy, translation to in vivo applications presents additional challenges. RF hyperthermia is already employed clinically for localized tumor treatments; however, achieving precise and homogeneous temperature control in deeper tissues remains complex.

Since the present study focused primarily on the effective concentration range around the IC50 value, the biological significance of the responses observed at sub-effective concentrations was not investigated in detail. The main objective was to evaluate whether RF exposure could influence the cytotoxic response of compound **4** near its effective dose range rather than to characterize the behavior of low-dose concentrations. Therefore, the findings observed at sub-effective concentrations should be interpreted with caution and warrant further investigation. Although variable responses were observed in the low-dose groups under RF exposure, these findings were consistently reproduced in three independent experiments performed in triplicate and were therefore retained in the analysis.

One limitation of the present study is the accuracy of the infrared temperature sensor used for routine monitoring. Future studies employing higher-precision temperature control systems may further improve the reproducibility and characterization of RF-induced hyperthermia. Factors such as tissue perfusion, heterogeneous thermal conductivity, and proximity to critical organs must be carefully managed to avoid off-target effects.

These considerations underscore the need for standardized RF dosimetry, real-time thermal monitoring, and predictive modeling of tissue heating to ensure safe and effective implementation in preclinical and clinical studies.

## 4. Materials and Methods

### 4.1. Chemistry

All solvents and reagents used in the present study were of analytical grade and utilized without further purification. Melting point was measured in open capillary tube using a Büchi B-540 apparatus (Flawil, Switzerland) and is uncorrected. Infrared spectrum (υ_max_, cm^−1^) was recorded in KBr disk on a Shimadzu IR Affinity-1 FTIR spectrophotometer (Kyoto, Japan). ^1^H and ^13^C NMR-APT spectra were recorded on Varian UNITY INOVA (500 MHz, Palo Alto, CA, USA) and Varian Mercury (Agilent, Santa Clara, CA, USA, 400 MHz) spectrometers using DMSO-d_6_ as solvent and TMS as internal standard. Chemical shifts (δ, ppm) and coupling constants (*J*, Hz) are reported. Mass spectrum (ESI) was obtained on a Waters 2695 Alliance Micromass ZQ LC/MS instrument, and elemental analysis was carried out on a Leco CHNS-932 analyzer (St. Joseph, MI, USA). Abbreviations: ar., aromatic; ind., indole; 2-ind., 2-indolinone; ph., phenyl.

#### 4.1.1. Synthesis of 1-(3-Methylbenzyl)-5-chloro-1*H*-indole-2,3-dione (**2**)

5-Chloro-1*H*-indole-2,3-dione (**1**) (5 mmol) and 10 mL of dimethylformamide (DMF) were stirred on a magnetic stirrer at room temperature for 1 h. The reaction mixture was heated to 50–60 °C and stirred for 2 h after the addition of potassium carbonate (K_2_CO_3_) (7 mmol), potassium iodide (KI) (1 mmol), and 3-methylbenzyl chloride (15 mmol), respectively. The resulting mixture was evaporated to dryness at low pressure. The solid product was washed with water to remove the excess of K_2_CO_3_ and KI, and a yellow amorphous solid was used without further purification. Yield: 98%; mp 158–159 °C [41,42,43].

#### 4.1.2. Synthesis of 1-(3-Methylbenzyl)-5-chloro-1,3-dihydro-2*H*-indole-2-one (**3**)

1-(3-Methylbenzyl)-5-chloro-1*H*-indole 2,3-dione (3.5 mmol) was heated under reflux (120–130 °C) in hydrazine hydrate (NH_2_NH_2_·H_2_O) (12 mL) for 4 h. The reaction mixture was cooled to room temperature and extracted with ethyl acetate. The residue obtained after the evaporation of ethyl acetate was treated with water and refrigerated overnight. A white amorphous solid product was filtered and used without further purification. Yield: 89%; mp 92–96 °C [44,45,46].

#### 4.1.3. Synthesis of 1-(3-Methylbenzyl)-3-(1*H*-indole-3-ylmethylidene)-5-chloro-1,3-dihydro-2*H*-indole-2-one (**4**)

1-(3-Methylbenzyl)-5-chloro-1,3-dihydro-2*H*-indole-2-one (3.5 mmol) was stirred under reflux (80–90 °C) with 1*H*-indole-3-carbaldehyde (4 mmol) in the medium of ethanol (20 mL) and 3 drops of piperidine for 5 h. After the reaction mixture was cooled, a yellow amorphous solid was filtered and purified by crystallization from a mixture of ethanol and chloroform [46,47,48].

#### 4.1.4. 1-(3-Methylbenzyl)-3-[(1*H*-indole-3-yl)methylidene]-5-chloro-1,3-dihydro-2*H*-indole-2-one (**4**)

Yellow amorphous (77%); mp 277–279 °C. IR (KBr) νmaks (cm^−1^): 3269 (N-H), 3062 (ar. C-H), 2920, 2852 (al. C-H), 1662 (C=O). ^1^H NMR (DMSO-d_6_/500 MHz) δ ppm: 2.26 (3H, s, ph. 3-CH_3_), 5.02 (2H, s, N-CH_2_C_6_H_5_), 6.92 (1H, d, *J*: 8.3 Hz, 2-ind. C_7_-H), 7.07 (1H, d, *J*: 7.5 Hz, ph. C_4_-H), 7.10 (1H, d, *J*: 7.8 Hz, ph. C_6_-H), 7.14 (1H, s, ph. C_2_-H), 7.18 (1H, dd, *J*: 8.3, 2.1 Hz, 2-ind. C_6_-H), 7.21 (1H, t, *J*: 7.5 Hz, ph. C_5_-H), 7.26–7.29 (2H, m, ind. C_5,6_-H), 7.54–7.57 (1H, m, ind. C_7_-H), 8.17 (1H, d, *J*: 2.1 Hz, 2-ind. C_4_-H), 8.29–8.33 (1H, m, ind. C_4_-H), 8.42 (1H, s, al. =CH), 9.56 (1H, s, ind. C_2_-H), 12.24 (1H, s, NH). ^13^C NMR-APT (DMSO-d_6_/125 MHz) δ ppm: 21.49 (ph. 3-CH_3_), 43.05 (N-CH_2_C_6_H_5_), 110.17 (2-ind. C_7_), 112.01 (ind. C_3_), 112.88 (ind. C_7_), 116.66 (2-ind. C_3_), 119.13 (2-ind. C_4_), 119.28 (ind. C_4_), 121.58 (ind. C_5_), 123.28 (ind. C_6_), 124.77 (ph. C_6_), 126.31 (2-ind. C_6_), 126.33 (2-ind. C_3a_), 127.20 (2-ind. C_5_), 128.87 (ind. C_3a_), 128.18 (ph. C_4_), 128.47 (ph. C_2_), 129.02 (ph. C_5_), 130.73 (al. =CH), 135.16 (ind. C_2_), 136.44 (ind. C_7a_), 137.35 (ph. C_3_), 138.29 (2-ind. C_7a_), 138.34 (ph. C_1_), 166.49 (C=O). MS (ESI (-)) *m*/*z* (%): 397.66 ([M-H]^−^, 100), 399,49 ([(M-H)+2]^−^, 28). Anal. Calcd for C_25_H_19_ClN_2_O.^1/4^H_2_O (403.38): C, 74.53; H, 4.83; N, 6.94. Found: C, 74.37; H, 5.01; N, 7.09.

### 4.2. In Vitro Biological Studies

#### 4.2.1. Cell Culture

In vitro experiments were conducted using the HCT116 human colon cancer cell line (ATCC^®^ CCL-247™) and the BEAS-2B human bronchial epithelial cell line (ATCC^®^ CRL-9609™), both obtained from the American Type Culture Collection (ATCC^®^, Manassas, VA, USA). Cells were cultured in Dulbecco’s Modified Eagle Medium (DMEM; Capricorn-Scientific) with glucose, supplemented with L-glutamine, 10% fetal bovine serum (FBS; Capricorn-Scientific, Ebsdorfergrund, Germany), and 100 U/mL penicillin and 100 μg/mL streptomycin (1% penicillin–streptomycin) (Capricorn-Scientific). Cultures were maintained under standard sterile conditions at 37 °C in a humidified atmosphere containing 5% CO_2_.

When cultures reached approximately 80–90% confluency, cells were washed with 1× PBS (Capricorn-Scientific) and detached using a trypsin (0.25%)–EDTA (0.02%) solution for sub-culturing. To minimize biological variability, all experiments were performed using cells between passages 7.

#### 4.2.2. Validation of the RF Hyperthermia Device, Design and Construction of the RF Hyperthermia Device

The mobile RF hyperthermia system was composed of two main units: an RF power generator and an impedance-matching unit. The RF generator served as the primary signal source and operated at a fixed frequency of 13.56 MHz with an adjustable output power range of 0–300 W. The frequency of 13.56 MHz was selected because it is an internationally recognized standard for biomedical use and allows for safe energy penetration through biological tissues.

The matching unit was integrated to ensure efficient, low-loss transmission of RF energy from the generator to the biological target. This unit is critical for minimizing signal reflection and electromagnetic interference, thereby preventing potential damage to adjacent electronic components. The RF generator and matching unit were connected through a specialized coaxial signal cable, while RF energy was delivered to the cell culture area via a silver-based high-conductivity coaxial cable to minimize energy loss during transmission. The hyperthermic effect arises from the oscillation of intracellular ions, such as water molecules, Ca^2+^, Zn^2+^, and other metallic ions, under alternating electromagnetic fields at 13.56 MHz. This rapid ionic movement leads to frictional heating within the cellular microenvironment. When the intracellular temperature exceeds 40 °C, the condition is referred to as hyperthermia, which is known to impair cellular functions and potentiate therapeutic effects. The extent of hyperthermia depends on the applied RF power and exposure duration. It is worth noting that 13.56 MHz RF systems can exert both thermal effects via ionic friction and non-thermal electromagnetic effects on cellular membranes. Since a purely convective heating control (such as a standard incubator) was not evaluated, the enhanced cytotoxicity observed in our study should be interpreted as a combined effect of RF-induced hyperthermia and RF exposure.

Preliminary calibration studies were performed by applying 40–100 W of RF power to the cell culture medium containing cancer cells. Depending on the initial medium volume and environmental conditions, exposure durations of 5–10 min were tested. A 40 W exposure for 5 min resulted in an average temperature increase of approximately 10 °C, confirming the device’s ability to induce controlled mild hyperthermia.

A custom sample chamber was fabricated using a 3D printer to accommodate standard culture flasks (25 cm^2^ or 75 cm^2^ cell culture flasks). Electrodes were embedded in the chamber to enable direct RF exposure, and a dual-point temperature monitoring system was incorporated to simultaneously measure internal (within the flask) and external temperatures during treatment.

#### 4.2.3. Drug Treatment and Application RF Hyperthermia Exposure

For combination treatments, compounds and RF exposure were applied simultaneously, with the compounds remaining present throughout the RF exposure period. Preliminary control experiments confirmed that both sunitinib and compound **4** were chemically stable under the applied RF conditions. HCT116 and BEAS-2B cells were seeded at 1 × 10^4^ cells per well in 96-well plates and incubated overnight at 37 °C prior to treatment. Cells were then treated with 0.625, 1.25, 2.5, 5.0, or 10.0 µM concentrations of each compound, either alone, under RF hyperthermia, or in combination. After treatment, cells were incubated at 37 °C for an additional 72 h before further analyses.

RF hyperthermia parameters were optimized based on prior calibration studies The 96-well plate containing cells was placed between square copper electrodes measuring 10 × 10 × 0.1 cm. The system, consisting of the copper electrodes and the 96-well plate structure, was embedded in polyurethane to isolate it from the external environment, thereby minimizing heat loss. Because RF signals can directly heat metal probes and cause inaccurate measurements during the RF exposure (due to electromagnetic wave–metal atom interactions), a non-contact infrared thermometer (Uni-T Ut 306S-50 °C to 500 °C, ±1.8 °C or ±1.8%) was used for temperature measurements. Temperature monitoring during RF exposure was performed using a contactless infrared thermometer (were performed in triplicate to ensure reproducibility). In addition, temperature measurements were validated by thermocouple measurements and thermal imaging analysis to ensure reliable assessment of temperature distribution and maintenance of hyperthermic conditions.

The thermal dose delivered during RF exposure can be approximated using the cumulative equivalent minutes at 43 °C (CEM43) model, which is commonly used to standardize hyperthermia treatments. Considering the exposure duration of 35 min and an average temperature of approximately 42 °C, the estimated CEM43 value can be calculated using the following equation:

CEM43 = t × R^(43 − T)^

where t is the exposure time (min), T is the average temperature (°C), and R = 0.25 for temperatures below 43 °C. Based on this approximation, the applied RF treatment corresponds to a thermal dose of approximately 8–9 min at 43 °C. This value falls within the typical mild hyperthermia range reported in previous studies investigating thermal sensitization of cancer cells.

Temperature measurements were performed using an infrared non-contact thermometer (−50 °C to 500 °C, ±1.8 °C or ±1.8%). Cellular viability and cytotoxic effects were evaluated by MTT assay. All temperature measurements were performed in triplicate.

Control and treatment groups were as follows:

Control 1: Untreated cells (no exposure)

Control 2: Cells exposed to RF hyperthermia only

Treatment group 1: Cells treated with compounds only

Treatment group 2: Cells treated with compounds + RF hyperthermia

#### 4.2.4. Cell Cytotoxicity Assay

The absorbance values of the cells were assessed by MTT assay (Sigma-Aldrich, St. Louis, MO, USA; Merck KGaA, Darmstadt, Germany). After all treatment for all groups, the medium was replaced by DMEM containing 0.5 mg/mL MTT and incubated for 2–4 h at 37 °C. The formazan crystals were dissolved using DMSO at room temperature. Thereafter, the optical density of the formazan solution was measured at 570 nm using an ELISA microplate reader (Allsheng FlexA-200, Hangzhou Allsheng Instruments Co., Ltd., Hangzhou, China).

#### 4.2.5. Flow Cytometric Analysis of Cell Death Using Annexin V/PI Staining

HCT116 cells were seeded in 6-well plates and allowed to adhere overnight under standard culture conditions (37 °C, 5% CO_2_). Cells were then treated with compound **4** at concentrations of 5 µM and 10 µM. RF hyperthermia was applied simultaneously using a 13.56 MHz RF system at 75 W for the specified exposure duration. Control cells received RF treatment alone. Following treatment, cells were harvested by trypsinization, washed twice with cold phosphate-buffered saline (PBS), and resuspended in 1× Annexin V binding buffer. Cell viability and death were assessed using an Annexin V-FITC/Propidium Iodide (PI) apoptosis detection kit (BioLegend, San Diego, CA, USA) according to the manufacturer’s instructions. Briefly, cells were incubated with Annexin V-FITC and PI for 15 min at room temperature in the dark. Flow cytometric analysis was performed using a Beckman Coulter Navios flow cytometer equipped with a 488 nm laser. A minimum of 10,000 events per sample were acquired. Data were analyzed using Navios EX Software v2.0, 5. Cell populations were classified as viable (Annexin V^−^/PI^−^) and total cell death (sum of Annexin V^+^/PI^−^, Annexin V^+^/PI^+^, and Annexin V^−^/PI^+^). Doublets and debris were excluded based on forward scatter (FSC) and side scatter (SSC) gating.

### 4.3. Statistical Analysis

The statistical analysis was performed using GraphPad Prism software, version 9.00 (San Diego, CA, USA). The results were analyzed by Student T-test (Student’s *t*-test) to compare statistical differences among groups. *p*-values of <0.05 were considered significant. Data are presented as mean ± SEM (n = 3 biological replicates).

## 5. Conclusions

RF hyperthermia can effectively and safely enhance the efficacy of chemotherapeutic agents while minimizing damage to surrounding healthy tissues when applied in combination with newly developed anticancer compounds. In this study, we demonstrated that combined exposure to RF hyperthermia and compound **4** was associated with reduced viability in HCT116 cells. The present findings indicate that RF exposure was associated with reduced cell viability in HCT116 cells treated with both sunitinib and compound **4**. Although some variability was observed in the response to compound **4** at lower concentrations, the overall results support the potential of RF-assisted treatment approaches and warrant further investigation under optimized experimental conditions.

## Data Availability

The original contributions presented in this study are included in the article/Supplementary Material. Further inquiries can be directed to the corresponding author.

## Supplementary Materials

The following supporting information can be downloaded at: https://www.mdpi.com/article/10.3390/ijms27135953/s1.
