# Supplementary material for: The Effect of Radiofrequency Exposure on the Cytotoxic Activity of Sunitinib and a Novel Sunitinib-Class Compound 4, in Colorectal Cancer Cells
Source: Int J Mol Sci. 2026 Jul 2;27(13):5953. doi: 10.3390/ijms27135953 (PMC13362021; doi:10.3390/ijms27135953)
Supplement: Supplementary file 1 [file ijms-27-05953-s001.zip › ijms-4340780-supplementary.pdf]

General Synthesis Scheme

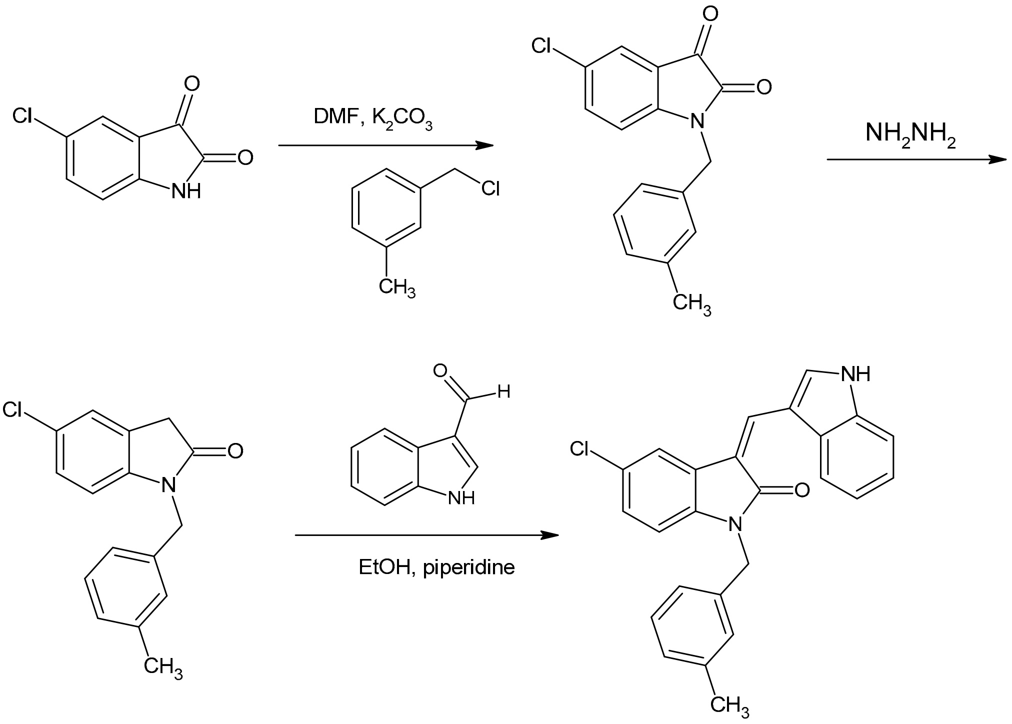

1-(3-Methylbenzyl)-3-[(1H-indole-3-yl)methylidene]-5-chloro-1,3-dihydro-2H-indole-2-one

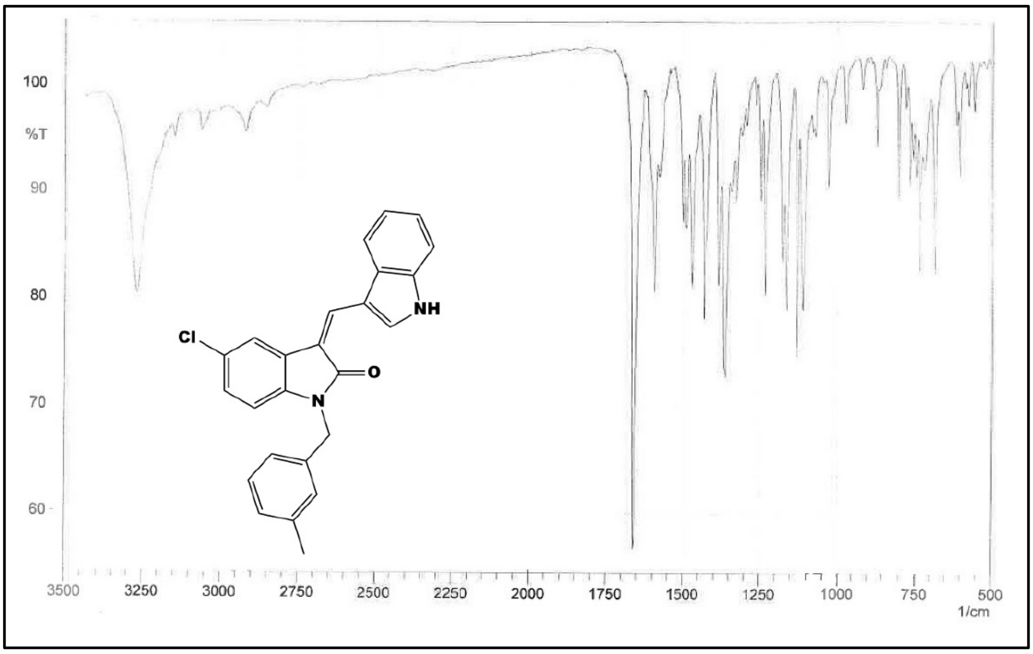

Figure S1. IR spectrum of compound **4**

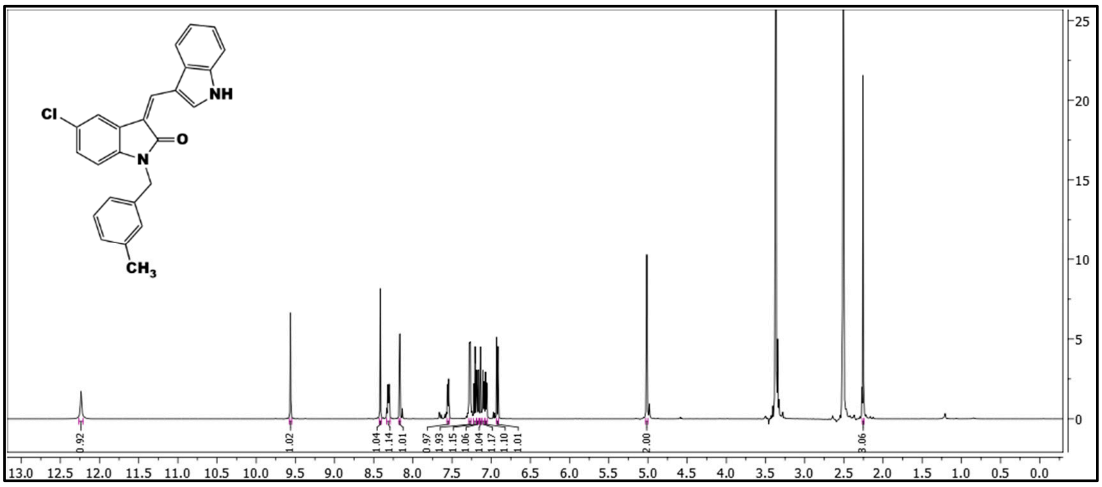

Figure S2. ^{1}H-NMR spectrum of compound **4**

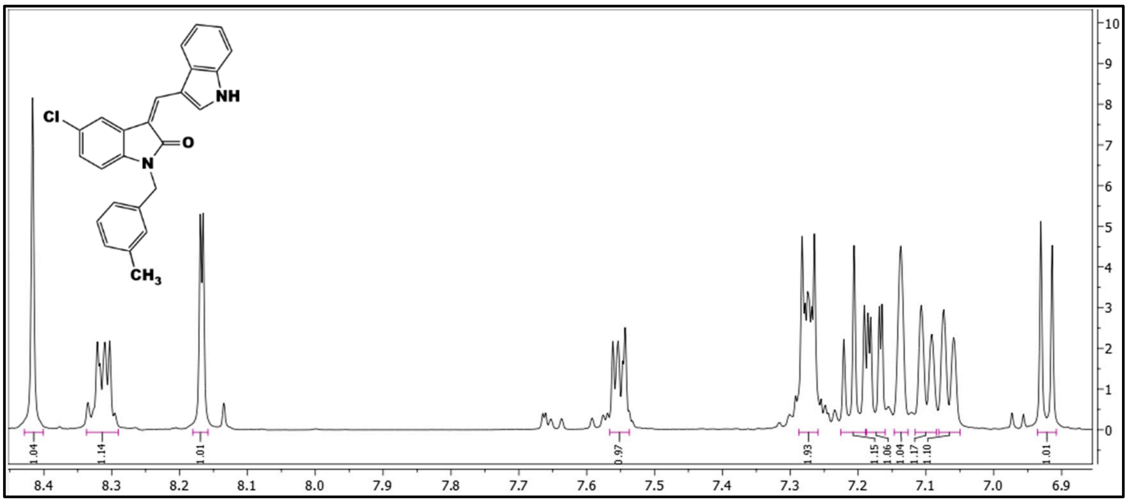

Figure S3. ^{1}H-NMR spectrum of compound **4**

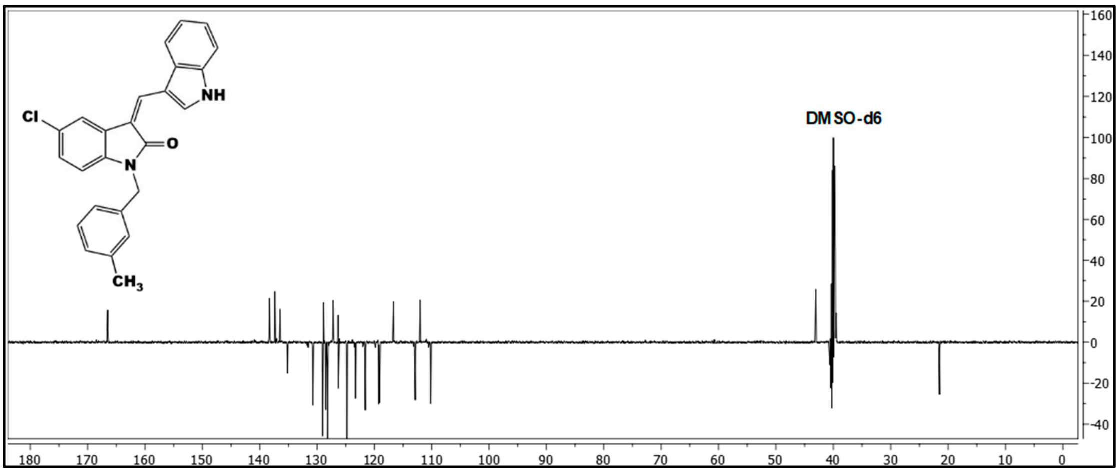

Figure S4. ^{13}C-NMR(APT) spectrum of compound **4**

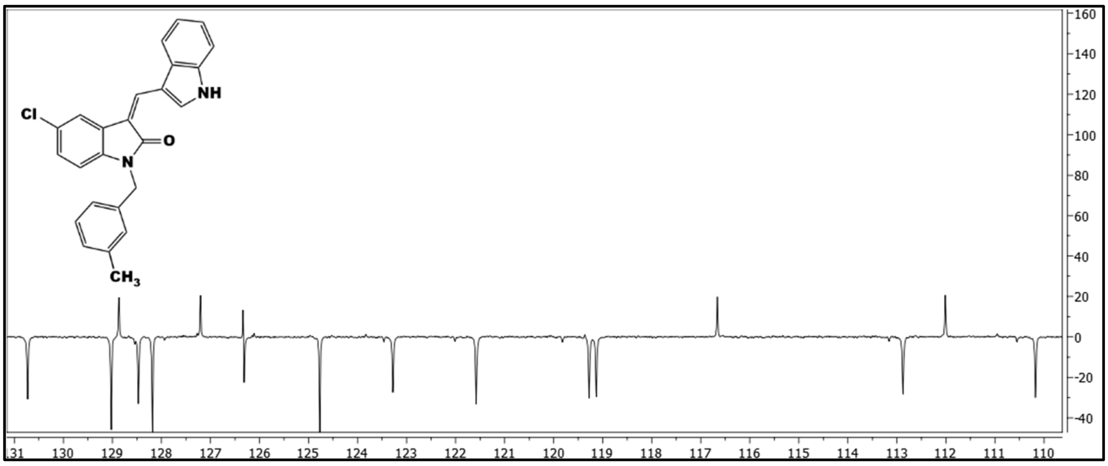

Figure S5. ^{13}C-NMR(APT) spectrum of compound **4**

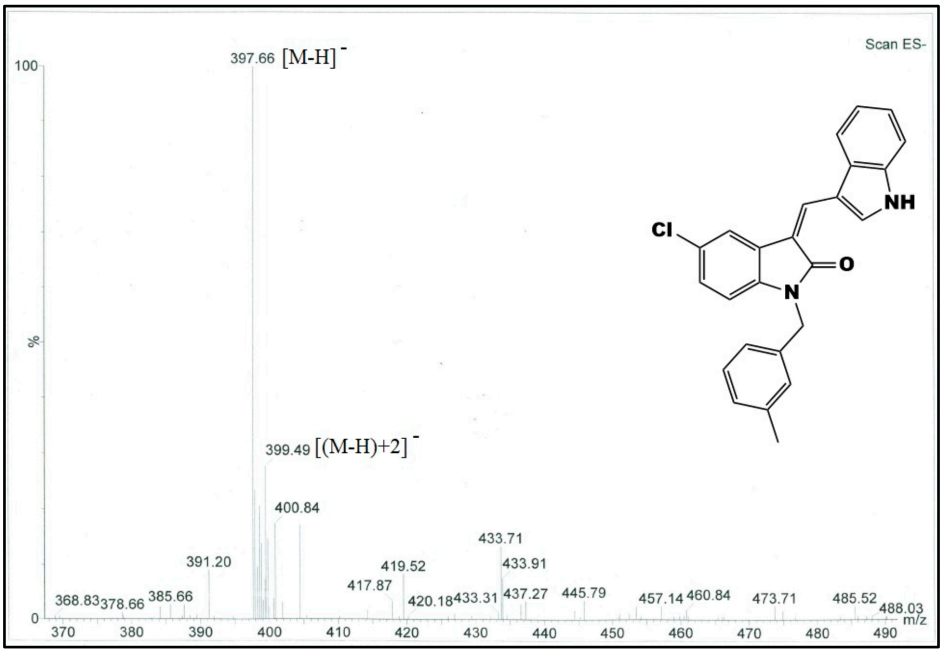

Figure S6. LC-MS spectrum of compound **4**
